# High School FLASH Sexual Health Education Curriculum: LGBTQ Inclusivity Strategies Reduce Homophobia and Transphobia

**DOI:** 10.1007/s11121-023-01517-1

**Published:** 2023-03-17

**Authors:** Kari Kesler, Andrea Gerber, BA Laris, Pamela Anderson, Elizabeth Baumler, Karin Coyle

**Affiliations:** 1 Public Health—Seattle & King County, Seattle, WA USA; 2 dfusion Inc, CA Scotts Valley, USA; 3 ETR, Scotts Valley, CA USA; 4 University of Texas Medical Branch Galveston, Galveston, USA

**Keywords:** LGBTQ-inclusive, Schools, Sexual health education, Adolescents, Curriculum development

## Abstract

Homophobic and transphobic beliefs that lead to bias-based harassment remain a critical concern for young people in the USA. The aim of the present study was to examine the impact of an inclusive comprehensive sex education program (High School FLASH) on homophobic and transphobic beliefs. Data from this study come from a randomized controlled trial that evaluated the impact of High School FLASH on students’ sexual behaviors and related outcomes with 20 schools in two U.S. regions (Midwest and South). Following the baseline survey, the 20 schools were randomly assigned to receive FLASH or a comparison curriculum. Ninth and 10th grade students completed follow-up surveys 3 and 12 months after the instructional period. We examined changes in homophobic beliefs using multilevel linear regression models in the full sample and two sub-groups: straight cisgender young people versus those who identified as not straight or cisgender. Mean scores on the homophobic and transphobic beliefs scale were statistically significantly lower among young people receiving FLASH relative to the comparison at both the 3- and 12-month timepoints (*p*-values for adjusted mean differences were < 0.01, *n* = 1357 and 1275, respectively). Specifically, FLASH’s positive impact on reducing homophobic and transphobic beliefs was statistically significant for straight and cisgender youth at both survey follow-ups (*p* < 0.01, *n* = 1144 and *p* = 0.05, *n* = 1078, respectively); the effects for the LGBTQ sub-group reached statistical significance at only the final follow-up (*p* = 0.01, *n* = 197). Our results show that carefully designed, inclusive comprehensive sexual health education programs like High School FLASH can play a role in promoting better school climates for all youth by reducing beliefs that may lead to bullying, violence, and victimization.

## Introduction

Homophobic and transphobic beliefs that lead to bias-based harassment remain a critical concern for young people in the USA. Homophobia and transphobia negatively impact people’s mental and physical health (Johns et al., 2019a, b; Proulx et al., 2019), leaving young people vulnerable to poor health outcomes across multiple domains, including sexual health (Hafeez et al., 2017; Johns et al., 2019a, b; Rasberry et al., 2018; Steinke et al., 2017). Lesbian, gay, bisexual, transgender, and queer (LGBTQ) young people report routinely hearing homophobic and transphobic language at school and experiencing discrimination and victimization related to their sexual orientation and/or gender identity (Kosciw et al., 2020), and a meta-analysis of 55 studies confirms that LGBTQ identification is a moderate and consistent risk factor for victimization at school (Myers et al., 2020). LGBTQ youth who are victimized and discriminated against in school face more negative academic consequences, including lower grade point average, absenteeism, disconnection from the school community, depression, and low levels of self-esteem, compared to LGBTQ youth who do not experience in-school victimization and discrimination (Kosciw et al., 2018).

This discrimination can, in part, be traced to the stigma and prejudice justified by pathologizing individuals who are not cisgender via diagnostic classifications (Suess Schwend, 2020). People have been forced to accept psychiatric diagnoses in order to access affirming care and related insurance benefits (Robles et al., 2021). The removal of diagnostic designations is a critical step to depathologize trans and nonbinary identities. While attempts have been made to begin depathologizing these identities, as evidenced by the modifications to the DSM diagnosis in 2022, the inclusion of the current diagnosis continues to pathologize all individuals who are not cisgender. Even as this work continues, schools can play an important role in addressing discrimination and transphobic violence against young people by adopting policies, programs, and training to support more inclusive environments.

Schools that create and enforce inclusive policies, adopt inclusive curricula, and sponsor gay-straight alliance (GSA) groups can improve school connectedness, which has been shown to improve academic, health, and well-being outcomes for young people (Snapp et al., 2015a, b; Day, 2020). Specifically, inclusive anti-bullying policies are associated with feelings of safety and less victimization (Kosciw et al., 2018; Russell et al., 2016). School-sponsored GSAs have been shown to improve the school climate, reduce victimization, and improve mental health for LGBTQ students (Day et al., 2020; Fetner & Elafros, 2015; Goodenow et al., 2006; Heck et al., 2013; Marx & Kettrey, 2016). Inclusive sex education that provides relevant information about sexuality and gender has been associated with positive mental health outcomes and a decrease in bullying and victimization (Proulx et al., 2019). Further, Snapp et al. (2015a, b) found that LGBTQ students who received inclusive sexual health curricula experienced lower levels of victimization, increased feelings of safety at school, fewer safety-related school absences, better academic performance, and increased feelings of connection to peers.

However, not all sex education programs that claim to be inclusive are designed in ways that affirm all young peoples’ identities and orientations. LGBTQ teens have identified a number of issues contributing to the lack of positive representation in their health curriculum (Gowen & Winges-Yanez, 2014), including silence on the part of the teacher or the curriculum about LGBTQ issues or individuals, heterosexism in the framing of the information presented, and pathologizing LGBTQ individuals or specific sexual practices. Despite the documented positive health outcomes achieved by offering young people inclusive school environments and curricula, few comprehensive sexual health programs have been designed intentionally to be relevant to all young people, underscoring the value of studying broader impacts of programs that are expressly designed as inclusive, such as the High School FLASH curriculum studied here.

### Curriculum Development 

High School FLASH is a comprehensive sexual health education curriculum developed and maintained by a county public health department. It is a public health strategy for classroom settings, with the specific behavior change goals of preventing unintended pregnancy, preventing STDs, and preventing the perpetration of sexual violence. FLASH is developed for use in public schools and as such is designed to be relevant and accessible to young people of all sexual orientations, genders, and family configurations. The Human Rights Campaign (HRC) states that LGBTQ inclusive sexual health education lessons are those that “help youth understand gender identity and sexual orientation with age-appropriate and medically accurate information; incorporate positive examples of LGBTQ individuals, romantic relationships and families; emphasize the need for protection during sex for people of all identities; and dispel common myths and stereotypes about behavior and identity” (HRC, 2021). Public health best practices were utilized to ensure FLASH is an inclusive and affirming curriculum, including an extensive process of pre-testing, piloting, and conducting key informant interviews with gatekeepers and end users before, during, and after lesson creation.

Inclusive content was created for use in all lessons, as well as creation of a lesson focusing specifically on the concepts of sexual orientation and gender identity. Messaging from all lessons was tested with a diverse group of young people, in which LGBTQ youth were purposefully overrepresented. Messages were adjusted according to feedback and re-tested until acceptability was reached. The lessons were subsequently piloted in public school classrooms to gauge understandability and ease of implementation. If revisions of any magnitude were required, they were again tested with groups of young people before being piloted once more. This process helped ensure that young people perceived the content to be affirming and relevant, while also ensuring it was understandable and promoted the behavioral outcomes of FLASH. Additionally, several strategies were tailored during the development of High School FLASH to create a curriculum that would reduce homophobia and transphobia (HRC, 2021; O’Farrell et al., 2021): (1) providing visibility; (2) affirming young people and families; (3) ensuring the relevance of content; and (4) using nuanced inclusive language. A description of each strategy and the processes used to tailor them are described below.

### Providing Visibility 

The first step toward improving inclusivity is to ensure young people can see themselves reflected in their curriculum (GLSEN, 2019). The young people characterized in scenarios, role plays, and vignettes in FLASH are depicted with a variety of sexual orientations and genders and in diverse contexts (e.g., sexually active, abstinent, partnered, single). Normalizing a wide range of identities, including those that are often dismissed or demeaned, demonstrates that all identities are valued, including those that might not be described specifically. Visibility is additionally reinforced using nuanced inclusive language (see below).

### Affirming Young People and Families 

Sex education instruction must additionally ensure that young people’s identities, and the identities of their friends and family, are being actively affirmed. To promote positive identity development young people must feel valued and respected. FLASH strives to affirm LGBTQ young people by portraying them in caring, satisfying, healthy relationships. While it is important to depict LGBTQ individuals in a variety of situations, including difficult situations, it is of the utmost importance to provide positive representations of LGBTQ young people and for students to see LGBTQ characters in healthy romantic relationships. In addition to affirming representations in the curriculum, teachers are instructed to use a specially designed protocol to affirm identities in class discussions and when answering questions, along all domains of identity (e.g., sexual orientation, gender, ability, religion, race, ethnicity).

### Ensuring Relevance of Content 

High School FLASH also works to ensure that the content of the curriculum is relevant for young people of all sexual orientations and genders by opting for forthright statements that provide visibility and create relevance with the existing content. For example, the birth control lesson in High School FLASH starts with the statement “this lesson is for everybody—people who are having vaginal sex now or who will in the future, and teens of all sexual orientations and genders. Even if someone won’t ever need birth control, learning about it now will help them act as health educators for their friends and families on this important topic.” This statement helps ensure the content feels relevant for young people who are having vaginal sex now or in the future, and those who may be having sex that can’t result in a pregnancy. An additional strategy used to create relevance (as well as improve accuracy) is to clearly state what sexual behaviors are being discussed at all times. Otherwise, young people may interpret “sex” to mean “only vaginal sex.” In addition to ensuring relevance, this approach supports the behavioral goals of the curriculum.

### Using Nuanced Inclusive Language 

High School FLASH uses a nuanced approach to inclusive language to strike a balance between broad inclusion and visibility of specific identities. The two approaches involve the use of neutral language, such as “partner,” as well as specific language, such as “boyfriend” or “girlfriend.” Each approach has advantages and disadvantages. Using both approaches, and applying each strategically, depending on context, allows for the most inclusive, affirming approach. We will discuss the disadvantages and complexities of these approaches in the discussion section.

Neutral language offers the advantage of a broad, welcoming umbrella. For example, the word “partner” is intended to mean a romantic partner of any gender, rather than the more specific “boyfriend” or “girlfriend.” Neutral language is intended to allow young people of any sexual orientation or gender to see themselves reflected, including individuals who may identify in ways that are unfamiliar to the teacher or curriculum author. Neutral language allows a single sentence or concept to be relevant to a large, diverse group of people. These are significant advantages.

The advantage of specificity is that people’s individual identities are named and provided visibility. Similarly, when specific genders and sexual orientations are named in scenarios all participants have the opportunity to consider what decisions young people like them might need to make. An ideal strategy is to use a mix of neutral and specific language to create a welcoming and affirming environment, and to listen carefully and be flexible when teaching so that a range of identities can be incorporated often.

While studies over the past decade have confirmed that inclusive school climates and offering inclusive sex education improve sexual health outcomes for both LGBTQ and non-LGBTQ youth (e.g., Pampati et al., 2021), as well as an array of other positive outcomes (Proulx et al., 2019), there is limited guidance on specific approaches to developing LGBTQ supportive school environments and inclusive curricula. Inclusive curricula aim to increase the visibility of LGBTQ people, experiences, and resources, and also to integrate critical thinking about how identities and realities are constructed (Page, 2017). Most existing guidance for creating an inclusive curriculum provides basic guidance to have zero-tolerance for homophobic comments, use inclusive language or create stand-alone lessons that include positive representation of LGBTQ people, history, and events (e.g., GLSEN, 2019). Inclusivity is a core component of High School FLASH. Although best practices to improve school climate for LGBTQ youth have been analyzed (Philbin et al., 2021), there has been little examination of the practices that lead to an actual decrease in homophobic and transphobic beliefs at the student level. This study seeks to extend the literature on the impact of inclusive sex education by examining the impact of High School FLASH on homophobic and transphobic beliefs.

## Methods

### Study Design

Data from this study come from the randomized controlled trial (NCT04079608) that evaluated the impact of High School FLASH on students’ sexual behaviors and related outcomes; the study involved 20 schools drawn from 7 districts in two regions (Midwest and South) in the USA (See Coyle et al. (2021) for full study description.) Following baseline data collection, the 20 schools were randomly assigned using computer-generated random numbers to receive the 15-session FLASH curriculum (*n* = 10 schools, 5 per region) or a 5-session knowledge-based sexual health curriculum (*n* = 10 schools, 5 per region) with a staggered start date for implementation (fall 2016 and fall 2017). The comparison curriculum was designed to increase health knowledge and was aligned to national health education standards with no specific LGBTQ inclusive strategies. All students within the participating ninth or 10th grade health education classrooms were invited to enroll. Follow-up surveys were administered 3 and 12 months after the instructional period. Final data collection ended in February 2019. The study procedures were reviewed and approved by the evaluators’ Institutional Review Board. Participating school districts also obtained approvals by their governing school boards. See Coyle et al. (2021) for a full study description.

### Study Participants

A total of 1597 students took part in the baseline survey (831 intervention and 766 comparison), representing 92% (1597/1734) of the students who had positive parent consent and were eligible for the primary study. (Consort diagram available in Coyle et al. (2021)). Follow-up surveys were administered in all study schools 3 and 12 months after curriculum implementation. The analytic sample for all outcomes in this paper includes all students who had a valid survey at the primary endpoint under consideration (3 or 12 months), which represents 1438 young people at the 3-month follow-up (750 intervention and 688 comparison) and 1395 young people at the 12-month follow-up (735 intervention and 660 comparison). Differential attrition was < 1% at the 3-month follow-up and 2% at the 12-month follow-up and overall attrition from baseline was 13% by the 12-month follow-up, which meets the low attrition standards for the What Works Clearinghouse.^1^ See Table 1 for more detail on the baseline and analytic samples.

**Table 1:** Demographics for sample

	**Overall**							**Non-queer**				**Queer**			
	Baseline sample (N = 1597)		3-month follow-up sample (N = 1438)			12-month follow-up sample (N = 1395)		3-month follow-up sample (n = 1211)		12-month follow-u sample (n = 1,177)		3-month follow-up sample (n = 227)		12-month follow-up sample (n = 218)	
	n	%	*n*		%	*n*	%	*n*	%	*n*	%	*n*	%	*n*	%
Gender															
Male	767	48	685		47.6	652	46.7	628	51.9	600	51	57	25.1	52	23.9
Female	808	50.6	732		50.9	724	51.9	580	47.9	574	48.8	152	67	150	68.8
Non-CIS	11	0.7	10		0.7	9	0.6	0	0	0	0	10	4.4	9	4.1
Missing	11	0.7	11		0.8	10	0.7	3	0.2	3	0.3	8	3.5	7	3.2
Race/ethnicity															
Asian	303	19	285	19.8		281	20.1	259	21.4	254	21.6	26	11.5	27	12.4
Black	659	41.3	592	41.2		571	40.9	519	42.9	502	42.7	73	32.2	69	31.7
White	350	21.9	324	22.5		314	22.5	250	20.6	245	20.8	74	32.6	69	31.7
Other	100	6.3	84	5.8		80	5.7	69	5.7	65	5.5	15	6.6	15	6.9
Multiracial	185	11.6	153	10.6		149	10.7	114	9.4	111	9.4	39	17.2	38	17.4
Mother was parent as teen	690	43.2	618	43		591	42.4	520	42.9	499	42.4	98	43.2	92	42.2
Primary language English	1,224	76.6	1,098	76.4		1,064	76.3	917	75.7	891	75.7	181	79.7	173	79.4
Mean age	15.3		15.3			15.3		15.3		15.3		15.3		15.3	

Sample size based on those with valid surveys at each time point. There are no statistically significant differences in overall demographic characteristics between the baseline sample and the analytic samples at 3- and 12-month follow-ups

### Data Collection Procedures

All surveys were voluntary and confidential. Trained data collectors administered the electronic self-report survey using tablets. Baseline data were collected before randomization in fall 2016 in the Midwest (872 youth in 55 classes in 10 schools) and fall 2017 in the South (725 youth in 40 classes in 10 schools). Baseline surveys were administered during health classes during the school day. Follow-up surveys were administered at school, pulling students from various classrooms because class schedules had changed. Students who left school after baseline were surveyed at their new schools, online, or by mail.

### Instrument

The student survey included items assessing demographic and cultural characteristics, sexual behavior, theory-based psychosocial factors, and program exposure. Survey items were selected based on the FLASH curriculum content and theory of change (Ajzen, 1991), and included items required by the funding agency. To reflect the LGBTQ inclusive nature of the curriculum, the survey instrument was also designed to be inclusive. For example, the survey provided nonbinary options for gender, comprehensive definitions of sexual behavior, inclusive terms for anatomy, and questions on puberty and the use of puberty blockers. The survey was available in English only. The survey was pilot tested with four classrooms of youth (*N* = 127) from three high schools in one region and one summer program in the other region prior to the implementation of the study to ensure readability, comprehension of terms, and improvement of the survey layout. The final survey took students approximately 30–40 min to complete.

### Study Measures

In this study, sexual orientation and gender identity were measured by two questions: “Which of the following best describes you?” (Response options: Female, Male, Trans Female, Trans Male, Gender Queer, Unknown, If none of these terms apply to you, please tell us how you describe your gender) and “Below is a list of terms that people often use to describe their sexuality or sexual orientation. Please check all those terms that apply to you.” (Response options: Gay, Lesbian, Bisexual, Straight/Heterosexual, Queer, Questioning, If none of these terms apply to you, please tell us how you describe your sexuality or sexual orientation.)

Homophobic and transphobic beliefs were assessed using a seven-item scale based on the Homophobic Belief Scale (Arseneau et al., 2013; Brownfield et al., 2018). Specific items included (1) it is okay for people to be gay, lesbian or bisexual; (2) you can tell someone’s sexual orientation by looking at them; (3) sexual orientation is an important part of a person’s identity; (4) I believe a person can be transgender; (5) I think all gay men act like women; (6) I think all lesbians act like men; and (7) I feel proud for rejecting stereotypes about people who are transgender. Response options ranged from 1 (strongly disagree) to 5 (strongly agree) with higher scores reflecting greater homophobic or transphobic beliefs (items 1, 3, 4, and 7 were reverse coded).

### Data Analysis

An intent-to-treat model was used in the analysis so that students were analyzed as randomized to treatment or comparison condition and followed up regardless of if they moved between schools. Multilevel linear regression models were used to assess treatment effects to account for non-independence of data from students sampled from the same school. Two-level models were fit with level-1 defined as the student and level-2 defined as the school. All models included an indicator variable denoting intervention group, the baseline outcome variable, a school size indicator, geographic region, and a set of a priori demographics (age, gender, race/ethnicity) as well as outcome-related covariates (mother was a parent as a teen, self-reported grades in school, importance of religion, number of guardians living in the home, self-reported maturity). Outcome related covariates were screened for potential model inclusion based on two criteria, associated with the study arm indicator variable at *p* < 0.15, and association with the outcome at *p* < 0.15 in individual bivariate analyses; two of those screened remained in all models (mother was a parent as a teen and importance of religion). Stata software version 16.1 was used for all analyses. For scaled variables, we calculated means scores for students who responded to at least 75% of the items included in a scale. Covariates represented baseline values. For covariates that were hypothesized to be stable over time (e.g., race and ethnicity or mother was a parent as a teen) we pulled data from the 3-month survey if they were available or from the 12-month survey if the variable was missing at baseline and 3 months; students with missing data on covariates in the models or who responded to fewer than 75% of the items in the belief scale were dropped from the analytic sample (*n* = 81 at 3-month follow-up and 120 at the 12-month follow-up), yielding 1357 participants in the final models for the 3-month follow-up and 1275 participants in the final models for the 12-month follow-up (shown in Tables 2 and 3). Models were fit both for overall as well as for subgroups of straight cisgender youth and of LGBTQ youth.

**Table 2:** Mean scores on the homophobic and transphobic belief scale at baseline, 3, and 12 months following random assignment

***First follow-up (3 months post curriculum implementation)***		
**Full sample (*N* = 1357)**	**Baseline mean (SD)**	**3-month follow-up mean (SD)**
Treatment	2.38 (0.67)	2.28 (0.69)
Comparison	2.34 (0.67)	2.33 (0.67)
**Straight & cisgender sub-group (*n*** **= 1144)**		
Treatment	2.43 (0.65)	2.33 (0.68)
Comparison	2.42 (0.65)	2.40 (0.64)
**LGBTQ sub-group (*n*** **= 213)**		
Treatment	2.11 (0.71)	1.99 (0.68)
Comparison	1.98 (0.63)	1.96 (0.69)
***Second follow-up (12 months post curriculum implementation)***		
**Full sample (*N*** **= 1275)**	**Baseline mean (SD)**	**12-month follow-up mean (SD)**
Treatment	2.38 (0.67)	2.26 (0.70)
Comparison	2.33 (0.67)	2.33 (0.68)
**Straight & cisgender sub-group (*n*** **= 1078)**		
Treatment	2.43 (0.65)	2.31 (0.69)
Comparison	2.41 (0.65)	2.39 (0.66)
**LGBTQ sub-group (*n*** **= 197)**		
Treatment	2.10 (0.72)	1.98 (0.69)
Comparison	1.93 (0.62)	2.06 (0.74)

Sample size based on those with valid surveys at each time point. Cases with missing values were excluded from the analyses (*n* = 81 at 3-month follow-up and 120 at the 12-month follow-up). Mean scores represent unadjusted values on a scale of 1 to 5 where higher values represent more homophobic or transphobic beliefs. Baseline characteristics of those excluded were similar to those in the starting analytic sample. There was no differential attrition between treatment conditions at 3 months. At 12 months, excluded cases from the FLASH condition were more likely to report English as a primary language than excluded cases from the comparison group; no other differences were present

**Table 3:** Adjusted mean comparisons on Homophobic and Transphobic Belief Scale at the 3- and 12-Month follow-up survey timepoints

**Time point**	**Sample**	***N***	**Beta (SE)**	***p*-value**	**ICC**
***3-month follow-up***	Full sample	1357	** − 0.072 (0.026)**	**0.005 < 0.01**	0.000
	Straight & cisgender sub-group	1144	** − 0.077(0.028)**	**0.005 < 0.01**	0.000
	LGBTQ sub-group	213	** − **0.021(0.065)	0.75	0.000
***12-month follow-up***	Full sample	1275	** − 0.076 (0.028)**	**0.008** **<0.01**	0.001
	Straight & cisgender sub-group	1078	** − 0.057 (0.030)**	**0.05**	0.000
	LGBTQ sub-group	197	** − 0.174 (0.068)**	**0.01**	0.000

Models adjusted for baseline value on belief scale, age, gender, region (Midwest or South), school size, race/ethnicity, time, religion, and report on whether students’ mother had children as a teen. The beta represents the difference in the adjusted means between treatment and control groups. Cases with missing values were excluded from the models (*n* = 81 at 3-month follow-up and 120 at the 12-month follow-up). Baseline characteristics of those excluded were similar to those in the starting analytic sample. There was no differential attrition between treatment conditions at 3 months. At 12 months, excluded cases from the FLASH condition were more likely to report English as a primary language than excluded cases from the comparison group; no other differences were present

## Results

Mean scores at baseline on the homophobic and transphobic beliefs scale were similar (2.38 among youth receiving FLASH versus 2.34 among those in the comparison condition) on a scale of 1 to 5 (SD = 0.67 for both groups), with higher means indicating more homophobic or transphobic beliefs. At the 3- and 12-month time points, mean scores improved among the group receiving FLASH (2.28 among youth in the treatment condition versus 2.33 among those in the comparison condition at the 3-month follow-up; 2.26 among youth in the treatment condition versus 2.33 among those in the comparison condition at the 12-month follow-up) (Table 2).

Comparisons of adjusted means from baseline to each follow-up show that High School FLASH reduced homophobic beliefs among the full sample and the effects were statistically significant at both survey follow-up time points (Table 3). Specifically, youth who received FLASH were less likely to endorse homophobic and transphobic beliefs relative to youth in the comparison condition who received a general knowledge-based curriculum (not FLASH) and the effects were evident at the short-term follow-up at 3 months after curriculum implementation (− 0.072 (0.026); *p* < 0.01, *n* = 1357) as well as the longer-term follow-up at 12 months after implementation (− 0.076 (0.028); *p* < 0.01, *n* = 1275).

FLASH’s positive impact on reducing homophobic and transphobic beliefs was noted for straight and cisgender youth as well as LGBTQ youth (Table 3), and the effects for the straight and cisgender sub-group reached statistical significance at both follow-up time points (*p* < 0.01, *n* = 1144 and *p* = 0.05, *n* = 1078, respectively). The effects for the LGBTQ sub-group reached statistical significance at final follow-up (*p* = 0.01, *n* = 197) but not the first follow-up (*p* = 0.75, *n* = 213).

## Discussion

This study found that High School FLASH reduced homophobic and transphobic beliefs for participating students when compared with a knowledge-only sexual health education curriculum. The reductions were significant though modest in magnitude as is common in prevention programs (Morales et al., 2018). The study results are meaningful on two levels: (1) they demonstrate that a school-based sexual health education program that effectively reduces the risk of unintended pregnancy and STDs can also decrease homophobia and transphobia, which has been shown to improve important physical health, mental health, and educational outcomes for young people (Johns et al., 2019a, b; Proulx et al., 2019; Myers et al., 2020); and (2) both LGBTQ participants and straight and cisgender participants experienced a reduction in phobic beliefs, which have different and important implications for each group. A reduction in homophobic and transphobic beliefs among LGBTQ students signals an improvement in how one feels about themselves (a decrease in internalized homophobia and transphobia), which is shown to improve mental and physical health (Amola & Grimmett, 2015; Gale et al., 2020). A reduction in homophobic and transphobic beliefs among straight and cisgender students reflects an improvement in how one perceives LGBTQ peers, which could potentially lead to a reduction in harassment and an improved school climate (theory of planned behavior [Ajzen, 1991]). FLASH is the first evidence-based teen pregnancy prevention program to date to report findings that show it reduces prejudice against people who are LGBTQ.

The results reported here, in conjunction with those shown in the outcome evaluation of FLASH (Coyle et al., 2021), are consistent with available studies showing that inclusive sexual health education has a positive impact on sexual health outcomes as well as a range of other important outcomes. For example, Blake et al. (2001) compared the outcomes for gay, lesbian, and bisexual youth at schools that provided “GLB-sensitive” prevention instruction to non-GLB-sensitive HIV instruction. They found that GLB students receiving the sensitive instruction were less likely to have had sex in the last 3 months, had fewer partners, and were less likely to have used substances the last time they had sex, as compared to their GLB peers who received no instruction or only minimally sensitive instruction. Proulx et al. (2019) found inclusive sex education decreased bullying and victimization, and Snapp et al. (2015a, b) found that LGBTQ students also experienced improved academic outcomes. The findings showing FLASH reduced homophobic and transphobic beliefs extend the literature on the value of implementing inclusive sex education programs and underscore the opportunity inclusive comprehensive sexual health education provides for strengthening the school climate, which is critically related to young people’s health, well-being, and academic success (U.S. Department of Education, Office of Safe and Healthy Students [USDE], 2016).

Although inclusive practices in sexual health education have been demonstrated to broadly yield an array of benefits for all youth (Proulx et al., 2019; Snapp et al., 2015a, b, we currently lack data on the impact of individual strategies. Further research is needed to explore which specific inclusivity strategies lead to these benefits and how benefits vary for young people based on sexual orientation and gender identity. Finally, this research should examine what strategies currently being used may have unintended negative consequences, so that well-intentioned educators do not inadvertently cause harm.

### Considerations for the Field

LGBTQ youth are largely unable to benefit from sexual health education to the same extent as their straight and cisgender peers because they frequently do not see themselves represented in the sexual health education they receive. A GLSEN study (2015) showed that less than 6% of LGBTQ students surveyed reported that their health class had included positive LGBTQ representation, while another survey of 18-–35-year-olds showed that only 12% of respondents ever had same-sex relationships mentioned in their health classes (Jones & Cox, 2015; Kosciw et al., 2016).

There is limited guidance or established best practices for developing new or adapting existing sexual health education curricula to make them inclusive of all genders and sexual orientations (Ioverno & Russell, 2022). Strategies used in FLASH are described in the introduction to this article. Applying these strategies is not without challenge, however, and program and research teams working to improve program inclusivity will benefit from a continuous quality improvement framework that prioritizes youth input. Without careful attention, attempts to improve inclusiveness can unintentionally cause harm by reinforcing stereotypes, exotifying or othering LGBTQ identities, and pathologizing LGBTQ individuals and any sexual behavior they may engage in (e.g., only mentioning LGBTQ identities in STD/HIV lessons; framing bisexual individual as having higher risk behaviors; and labeling characters as LGBTQ but failing to label the identities of straight and cisgender characters) (Formby, 2015; Gowen et al., 2013; Harris et al., 2022). Curriculum developers will benefit from understanding these potential unintended consequences. These risks can be mitigated by collecting specific feedback during the creation of the lessons, during piloting, and during implementation.

Ensuring relevance is an especially important strategy since LGBTQ teens are at an increased risk for unintended pregnancy and other poor sexual health outcomes (Hafeez et al., 2017; Johns et al., 2019a, b; Rasberry et al., 2018; Steinke et al., 2017). Because sexual behavior and sexual orientation do not always perfectly align, and because of additional risk factors that are overrepresented among LGBTQ teens, sexual health information needs to be relevant and accessible to all young people, even if it is not immediately pertinent. A common but problematic strategy to address relevance in sexual health education is to overstate the relative risk of sexual activities. For example, representing the risk of oral sex on a vulva as if it carries the same risk as vaginal sex overstates the relative risk (CDC, n.d.). The intent is likely to provide relevant content by addressing oral sex. However, pathologizing lower-risk sexual practices is disingenuous and is an ineffective strategy for ensuring relevance.

Using nuanced, inclusive language also requires careful reflection, continual refinement, input from youth, and careful attention to literacy levels. For example, when discussing birth control methods that suppress ovulation, a neutral approach would state that these methods are “for a person who has ovaries,” rather than stating that they are for women. The advantage of this is that it is broad and non-exclusive. Any person with ovaries can use existing hormonal birth control methods unless they have been medically advised not to. However, there are drawbacks to this approach. “Person with ovaries” requires much higher literacy to comprehend than “woman.” Simply stating woman, however, leaves some people out. Additionally, “person with ovaries” or “person with testicles” may be seen as objectifying, identifying a person by the parts of their body. The approach studied here utilized a mix of strategies. Birth control lessons describe a “person with ovaries” when discussing hormonal birth control and describe a “condom for a penis” or a “condom for the vagina or anus” when discussing condoms. However, the introduction to the reproductive system lesson states that “these parts are usually on a man's body,” or are “usually on a woman’s body” to provide needed context to students with lower health literacy.

Another disadvantage of neutral language is that it does not provide visibility. Specifically, individuals whose identities have been marginalized are unlikely to see themselves reflected in neutral language. For example, educators may use neutral language to explain that the content is for “all young people” instead of using specific language to explain that the content is for “lesbian, gay, bisexual, and straight young people.” Lesbian, gay, and bisexual young people will likely not assume the content is relevant to them, despite the teacher’s attempt to welcome all young people. Conversely, a drawback of specific language is that it fails to be fully inclusive. The term “LGBTQ” is specific only to five identities. The terms people use to describe their identities are personal, numerous, and evolving. These points illustrate that approaches to inclusivity have advantages and disadvantages; there is no universal fit.

An additional drawback to the use of neutral language involves a specific application. An approach sometimes used in the field is to construct neutral scenarios that do not specify the gender of the characters and use gender-neutral names such as Chris. This strategy has significant drawbacks. Young people will likely assume “Chris” to be a straight cisgender person, negating any potential benefit. Neutral language is sometimes used in this way when the educator is uncomfortable naming the specific identity, believes others will find it offensive, or believes the climate to be hostile. In these situations, use of neutral language can reinforce the idea that LGBTQ identities are taboo. Educators should exercise caution to ensure they are not using neutral language in this way.

The challenges reflect, in part, the dynamic nature of language and of identities. An approach that is perceived as affirming and inclusive at one time will not always be experienced in that way. Teachers who are adept at providing inclusive instruction are aware that language is constantly changing. Educators would benefit from being aware of these issues and developing these skills in the sex ed classroom, as they do in other subject areas.

Finally, teachers must be provided adequate training to implement these strategies and to be prepared to answer young people’s questions. There are skills related to the use of neutral and specific language, to creating relevance, and to affirming young people’s identities that are not often part of teachers’ professional development. In their meta-analysis of the literature on LGBTQ-inclusive sex ed, O’Farrell et al. (2021) speaks to challenges faced by teachers. They state that there was “a reported ambivalence and anxiety on the part of some facilitators to deliver inclusive sexual health education due to their own inherent stigma or perceived inability to address such topics due to lack of information and training. In fact, training and lack of information/resources to help in the delivery of sexual health was the main reason facilitators identified as to why they felt unable and ill-equipped to deliver LGBTQ + inclusive sexual health education.”

Educators need training that helps them broadly understand the concepts of sexual orientation, gender identity, assigned sex, and sexual behavior. Basic terminology should be covered with an understanding that language is always evolving, and that young people have the right to self-identify. Teachers need an opportunity to practice using key concepts and answering difficult questions using strategies that maintain an affirming environment for all young people in their classrooms.

### Limitations

The sample size of LGBTQ youth was relatively small, limiting our ability to examine the data with more refined sub-groups. Further, this study represents two regions in the U.S. and data may not generalize to other regions. Nonetheless, the resulting data provide new insights regarding the potential of high-quality inclusive sexual health education curriculum on reducing homophobic and transphobic beliefs.

## Conclusions

High School FLASH showed a short- and longer-term impact on reducing homophobic and transphobic beliefs among both LGBTQ and straight and cisgender high school youth. The results show that carefully designed, inclusive comprehensive sexual health education programs such as FLASH play a critical role in promoting better school climates for all youth by reducing beliefs that may lead to bullying, violence, and victimization. Furthermore, the reduction in these beliefs among LGBTQ students suggest a decrease in internalized homophobia and transphobia, which has been shown to improve mental, physical, and sexual health. The use of tailored inclusivity strategies can create sexual health education that is relevant and affirming for all young people, improving sexual health outcomes overall.

## Data Availability

Supporting data for this study are not publicly available due to participant consent restrictions.

## Footnotes

1 Attrition from total eligible (1734) to the 12-month follow-up (1395) was 19.55%.
